# Reduced Independence in Daily Living Is Associated with the Gut Microbiome in People with HIV and HCV

**DOI:** 10.1128/mSystems.00528-20

**Published:** 2020-10-13

**Authors:** Bryn C. Taylor, Kelly C. Weldon, Ronald J. Ellis, Donald Franklin, Daniel McDonald, Gregory Humphrey, MacKenzie Bryant, Julia Toronczak, Tara Schwartz, Jennifer Iudicello, Robert Heaton, Igor Grant, Sara Gianella, Scott Letendre, Austin Swafford, Pieter C. Dorrestein, Rob Knight

**Affiliations:** a Biomedical Sciences Graduate Program, University of California San Diego, La Jolla, California, USA; b Collaborative Mass Spectrometry Innovation Center, Skaggs School of Pharmacy and Pharmaceutical Sciences, University of California San Diego, La Jolla, California, USA; c Center for Microbiome Innovation, University of California San Diego, La Jolla, California, USA; d Department of Neurosciences, HIV Neurobehavioral Research Center, University of California San Diego, La Jolla, California, USA; e Department of Psychiatry, HIV Neurobehavioral Research Center, University of California San Diego, La Jolla, California, USA; f Department of Psychiatry, School of Medicine, University of California San Diego, La Jolla, California, USA; g Department of Pediatrics, School of Medicine, University of California San Diego, La Jolla, California, USA; h Division of Infectious Diseases and Global Public Health, University of California San Diego, La Jolla, California, USA; i Department of Medicine, University of California San Diego, La Jolla, California, USA; j Department of Psychiatry, University of California San Diego, La Jolla, California, USA; k Department of Computer Science and Engineering, University of California San Diego, La Jolla, California, USA; l Department of Bioengineering, University of California San Diego, La Jolla, California, USA; University of Pennsylvania

**Keywords:** IADL, gut microbiome, gut-brain axis, human immunodeficiency virus, hepatitis C virus

## Abstract

Alterations in the gut microbiome are associated with neurocognition and related disorders, including in the context of human immunodeficiency virus (HIV) and hepatitis C virus (HCV) infection. However, the connection between the gut microbiome and cognitive decline, gauged by increased dependence in instrumental activities of daily living (IADL), remains largely unexplored in the context of these diseases. Here we characterized the gut microbiome using 16S rRNA amplicon sequencing and untargeted metabolomics with liquid chromatography-mass spectrometry from 347 people with HIV, HIV and HCV, or neither, all of whom underwent a comprehensive neuropsychiatric assessment. We observed that IADL-dependent and -independent HIV-monoinfected (HIV-positive [HIV+]/HCV-negative [HCV−]) and coinfected (HIV+/HCV+) individuals have distinct gut microbiomes. Moreover, we found that dependent individuals with HIV or HIV and HCV were enriched in *Bacteroides*. These results may have implications for the characterization of cognitive decline, as well as the development of potential prevention and treatment strategies for individuals infected with HIV and/or HCV. Of particular interest is the possibility that dietary interventions that are known to modify the microbiome could be used to shift the microbiome toward more favorable states for preserving independence.

**IMPORTANCE** The microbes in the gut and the chemicals they produce by metabolism have been linked to brain function. In earlier work, we showed that infection with two viruses, HIV and HCV, changed the gut microbes and metabolism in ways that were associated with a lifetime history of major depressive disorder. Here, we extend this analysis looking at a measurement of independence in daily living. We find that in individuals with HIV, whether or not they also have HCV, those who reported reduced independence were enriched in a genus of bacteria called *Bacteroides*. This result is interesting because *Bacteroides* is strongly associated with diets low in carbohydrates and high in animal protein, suggesting that diet changes may help preserve independent living in people living long-term with HIV (although clinical intervention trials would be needed in order to confirm this).

## Abstract (precis)

The microbes in the gut and the chemicals they produce by metabolism have been linked to brain function. In earlier work, we showed that infection with two viruses, HIV and HCV, changed the gut microbes and metabolism in ways that were associated with a lifetime history of major depressive disorder. Here, we extend this analysis looking at a measurement of independence in daily living. We find that in individuals with HIV, whether or not they also have HCV, those who reported reduced independence were enriched in a genus of bacteria called *Bacteroides*. This result is interesting because *Bacteroides* is strongly associated with diets low in carbohydrates and high in animal protein, suggesting that diet changes may help preserve independent living in people living long-term with HIV (although clinical intervention trials would be needed in order to confirm this).

## OBSERVATION

Aging is associated with cognitive decline and increased risk of dementia. Antiretroviral therapy (ART) extends the life span of HIV-positive (HIV+) individuals, but even adequately treated HIV increases risk for these conditions. Early signs of cognitive decline include impaired instrumental activities of daily living (IADL) and increased aging in HIV+ individuals (1, 2). Increased risk for impaired IADLs is greatest with lower CD4 counts or AIDS onset. Impaired ADL and IADLs are also associated with increased mortality (1, 3). Hepatitis C virus (HCV)-induced liver injury, a leading cause of mortality in HIV+ individuals, increases risk for impaired IADLs (4). More than 2,000,000 individuals worldwide are coinfected with HIV and HCV, and coinfection is particularly common in developed Western countries (5). Coinfected (HIV+/HCV+) individuals use more drugs, a known risk factor for acquiring HCV and substance-induced major depression. Coinfected individuals have exhibited impaired executive functioning, independent of liver disease severity (6). The gut microbiome is altered in individuals with HIV (7) and HCV (8), and we recently linked HIV- and HCV-associated changes in the gut microbiome and metabolome with major depressive disorder (9). Extending this work, we tested whether the gut microbiome and metabolome in HIV-HCV coinfection are also linked to changes in IADL.

We used a previously characterized group (9) of 398 participants with or without HIV or HCV infection from the University of California (UC) San Diego HIV Neurobehavioral Research Program, who underwent standardized evaluation of neuropsychiatric diagnoses using *Diagnostic and Statistical Manual of Mental Disorders*, 4th edition (DSM-IV) (10) criteria (see “Methods” below) and provided fecal samples. A total of 347 samples from unique participants remained after applying filtering and exclusion criteria (see “Methods” below) and retaining only participants who reported their IADL status. Participants were grouped according to HIV and HCV infection status for analyses to assess the correlation of their daily living ability (IADL subgroups of “dependent” or “independent”; see “Methods” below; Table 1).

**TABLE 1:** Demographic information of the full cohort studied for the relationship between HIV, HCV, IADL, and the gut microbiome^*a*^

Characteristic^*b*^	Value for the following subgroup^*c*^:								
	Uninfected (HIV−/HCV−)			HIV-monoinfected (HIV+/HCV−)			Coinfected (HIV+/HCV+)		
	IADL dep.	IADL indep.	*P* value	IADL dep.	IADL indep.	*P* value	IADL dep.	IADL indep.	*P* value
*n*	10	91		73	127		22	24	
Age (yr)^*d*^	48.8 (18.4)	51.3 (16.4)		54.7 (11.1)	50.0 (12.2)	0.008	54.7 (10.6)	53.0 (8.1)	
Education (yr)^*d*^	13.3 (2.8)	14.6 (2.5)		14.1 (2.5)	14.2 (2.4)		13.4 (2.7)	13.7 (2.6)	
Gender									
% male	60	60		86	87		77	92	
% female	40	40		14	13		23	8	
Ethnicity									
% African American	20	19		18	19		36	33	
% Caucasian	40	59		62	56		32	46	
% Hispanic	20	20		16	20		32	21	
% other	20	2		4	5		0	0	
Estimated verbal IQ^*d*^	99.2 (19.5)	105.6 (15.3)		102.2 (12.9)	101.8 (12.6)		100.5 (16.0)	98.2 (11.8)	
Sexual orientation (%)									
Bisexual	0	8		6	10		23	13	
Heterosexual	70	70		23	18		27	26	
Homosexual	30	22		70	71		50	61	
Other/not asked				1	1				
% AIDS				70	52	0.0164	77	67	
Estimated duration of infection (yr)^*d*^				19.9 (9.3)	16.4 (10.0)	0.0183	21.6 (8.6)	22.0 (6.7)	
Nadir CD4^*e*^				135 [13–246]	200 [43–352]	0.0281	169 [17–254]	112 [7–284]	
Current CD4^*e*^				617 [452–902]	638 [482–829]		480 [295–796]	588 [456–848]	
% undetectable plasma on ART				87	88		72	94	
% undetectable CSF on ART				96	89		82	100	
ART status									
% HAART				96	97		86	88	
% off ARVS				4.0	1.5		14.0	8.0	
% ARV naive				0.0	1.5		0.0	4.0	
% employed	22	46		16	43	0.0002	0	35	0.004
Karnofsky disability rating	89 (9.9)	98.0 (6.8)		84.3 (14.3)	95.4 (8.0)	<0.0001	73.5 (16.0)	92 (12.4)	0.0002
BDI-II^*d*^	13 (10.6)	4.9 (6.5)	0.0013	16.2 (12.2)	7.9 (8.4)	<0.0001	16.4 (11.5)	6 (7.2)	0.002
GDS	0.77 (0.50)	0.54 (0.53)		0.64 (0.49)	0.56 (0.54)		0.86 (0.68)	0.58 (0.59)	
GDS impairment (%)	75	39	0.049	56	45		68	43	
MDD (%)									
Lifetime	40	32		58	50		86	58	
Current	10	2		9	4		10	0	
Any substance Dx (%)									
Lifetime	70	51		74	73		91	83	
Current	10	10		7	7		15	5	

a Student’s *t* tests were used for all normally distributed continuous variables (age, education, estimated verbal IQ, estimated duration of HIV infection, and GDS). Wilcoxon tests were used for nadir CD4, current CD4, BDI-II, and Karnofsky disability rating. Chi-square tests were used for all nominal variables (percent Caucasian, percent AIDS, percent undetectable plasma on ART, percent undetectable CSF on ART, percent bisexual and/or homosexual, ART status, percent employed, lifetime MDD, lifetime methamphetamine Dx, lifetime sedative Dx, and lifetime any substance Dx). The *P* value column for each infection group indicates whether the IADL groups within that infection type show a significant difference (alpha = 0.05).

b ART, antiretroviral therapy; CSF, cerebrospinal fluid; HAART, highly active antiretroviral therapy; ARV, antiretroviral; BDI-II, Beck Depression Inventory-II; GDS, global deficit score; MDD, major depressive disorder; Dx, substance use disorder.

c dep., dependent; indep., independent.

d Mean (standard deviation) shown for these characteristics.

e Median [interquartile range {IQR}] shown for these characteristics.

Dependent and independent individuals within each infection group are compared in Table 1. Gender and sexual practices in HIV patients may independently lead to differences in the gut microbiome (11), so we conducted a secondary analysis limited to men who have sex with men (MSM) (Table 2). Across all infection groups in the full cohort, we ran linear regressions for continuous variables and logistic regressions for categorical variables to look at the effects of IADL status on variables of interest. IADL dependence was significantly associated with greater neurocognitive impairment (higher continuous global deficit score [GDS], *P* = 0.03, and global impairment rate, *P* = 0.0043), unemployment (*P* < 0.0001), more clinician-rated disability (lower Karnofsky rating, *P* < 0.0001), more currently depressed mood (Beck Depression Inventory-II [BDI-II] score, *P* < 0.0001), and higher rates of DSM-IV diagnoses of lifetime (*P* = 0.038) and current (*P* = 0.023) major depressive disorder (MDD), and methamphetamine use disorder (DSM-IV diagnoses, *P* = 0.0005). However, IADL dependence was not significantly related to demographics (age, education, sex, race/ethnicity, and MSM status) or estimated premorbid verbal intelligence quotient (IQ) (based upon Wide Range Achievement Test-IV reading level). The pattern of these findings strongly supports that the self-reports of increased IADL dependence in these groups represent acquired central nervous system compromise, likely due to multiple causes (HIV/HCV infections, prior methamphetamine use disorders, and affective illness).

**TABLE 2:** Demographic information of the MSM subgroups studied for the relationship between HIV, HCV, IADL, and the gut microbiome^*a*^

Characteristic^*b*^	Value for the following subgroup^*c*^:								
	Uninfected (HIV−/HCV−)			HIV-monoinfected (HIV+/HCV−)			Coinfected (HIV+/HCV+)		
	IADL dep.	IADL indep.	*P* value	IADL dep.	IADL indep.	*P* value	IADL dep.	IADL indep.	*P* value
*n*	3	22		52	99		16	17	
Age (yr)^*d*^	43 (31.3)	54.1 (15.0)		53.2 (11.7)	50.2 (13.2)		53.6 (11.6)	52 (6.6)	
Education (yr)^*d*^	15 (2.6)	15.0 (2.4)		14.4 (2.5)	14.5 (2.4)		14.0 (2.8)	13.2 (2.3)	
Ethnicity									
% African American	0	0.14		0.12	0.12		0.25	0.24	
% Caucasian	34	68		65	64		38	53	
% Hispanic	33	14		19	20		37	23	
% other	33	4		4	4		0	0	
Estimated verbal IQ^*d*^	111.3 (14.6)	110.6 (18.1)		103.9 (11.8)	102.5 (11.4)		103.8 (17.1)	100.7 (10.0)	
Sexual orientation (%)									
Bisexual	0	23		8	11		31	18	
Homosexual	100	77		92	89		69	82	
% AIDS				69	49	0.014	75	65	
Estimated duration of infection (yr)^*d*^				20.6 (9.8)	16.5 (10.5)	0.0245	21.9 (9.5)	23.0 (6.9)	
Nadir CD4^*e*^				135 [20–250]	209 [79–400]	0.0237	178 [22–458]	114 [9–298]	
Current CD4^*e*^				612 [448–910]	643 [473–820]		405 [268–725]	529 [455–828]	
% undetectable plasma on ART				84	87		77	100	
% undetectable CSF on ART				100	10		88	100	
ART status									
% HAART				94	96		88	88	
% off ARVS				6.0	2.0		12.0	6.0	
% ARV naive				0.0	2.0		0.0	6.0	
% employed	33	38		13	43	0.0003	0	44	0.0047
Karnofsky disability rating	90 (10)	96.2 (11.2)		83.6 (14.6)	95.4 (8.0)	<0.0001	74.7 (17.7)	92.3 (11.7)	0.005
BDI-II^*c*^	12 (7.5)	6 (6.8)		16.5 (12.6)	8.3 (8.9)	<0.0001	15.1 (12.0)	5.2 (4.8)	0.0135
GDS	0.57 (0.45)	0.56 (0.51)		0.61 (0.43)	0.57 (0.56)		0.95 (0.73)	0.57 (0.64)	
GDS impairment (%)	67	38		53	47		71	38	
MDD									
Lifetime (%)	33	32		54	51		81	47	0.0413
Current	0	0		8	4		13	0	
Any substance Dx (%)									
Lifetime	67	77		77	75		88	88	
Current	0	14		4	7		13	0	

a Student’s *t* tests were used for all normally distributed continuous variables (age, education, estimated verbal IQ, estimated duration of HIV infection, and GDS). Wilcoxon tests were used for nadir CD4, current CD4, BDI-II, and Karnofsky disability rating. Chi-square tests were used for all nominal variables (percent Caucasian, percent AIDS, percent undetectable plasma on ART, percent undetectable CSF on ART, percent bisexual and/or homosexual, ART status, percent employed, lifetime MDD, lifetime methamphetamine Dx, lifetime sedative Dx, and lifetime any substance Dx). The *P* value column in each infection group, *P* value, indicates whether the IADL groups within that infection type show a significant difference (alpha = 0.05).

b ART, antiretroviral therapy; CSF, cerebrospinal fluid; HAART, highly active antiretroviral therapy; ARV, antiretroviral; BDI-II, Beck Depression Inventory-II; GDS, global deficit score; MDD, major depressive disorder; Dx, substance use disorder.

c dep., dependent; indep., independent.

d Mean (standard deviation) shown for these characteristics.

e Median [interquartile range {IQR}] shown for these characteristics.

Because neurobehavioral issues may result from altered gut microbiome composition and/or function, we tested for changes in overall gut microbiome diversity, assessed by 16S rRNA amplicon sequencing. In a subset of samples, the metabolome profile was assessed using untargeted liquid chromatography followed by tandem mass spectrometry. In the gut metabolome, no beta diversity (i.e., between-subject, Bray-Curtis) differences were seen between IADL states in any of the infection groups (see Table S2 in the supplemental material). The gut metabolome was analyzed using a partial least-squares discriminant analysis to identify potential features of interest. Further inspection of individual features indicated no significant differences between IADL status in any infection groups for any class of compounds. We also did not observe community-wide microbiome differences between IADL states within the uninfected full cohort and MSM subgroup, as neither beta diversity (unweighted UniFrac; full cohort, permutational analysis of variance [PERMANOVA] pseudo-F-statistic [pseudo-F] = 1.074, Benjamini-Hochberg-corrected [BH] *P* = 0.30; MSM subgroup, PERMANOVA pseudo-F = 0.92, BH *P* = 0.55) nor alpha diversity (i.e., within subject; Faith’s phylogenetic diversity [PD]; full cohort, Kruskal-Wallis [K-W] H = 0.058, BH *P* = 0.81; MSM subgroup, K-W H = 0.028, BH *P* = 0.87) differed between dependent (full cohort, *n* = 7; MSM subgroup, *n* = 22) and independent (full cohort, *n* = 94; MSM subgroup, *n* = 3) individuals. However, there were few dependent individuals, so the chance of type II error is high.

In contrast, in each of the infection groups and MSM subgroups, we observed significant community-wide microbiome differences between dependent and independent individuals. In the HIV-monoinfected (HIV+/HCV-negative [HCV−]) groups, there was a difference in beta diversity (unweighted UniFrac;. full cohort, PERMANOVA pseudo-F = 1.72, BH *P* = 0.02; MSM subgroup, pseudo-F = 1.56, BH *P* = 0.034), but not in alpha diversity, between the IADL groups (Faith’s PD; full data set, K-W H = 2.79, BH *P* = 0.095; MSM data set, K-W H = 0.93, *P* = 0.34). In the coinfected groups, beta diversity differed between independent and dependent individuals (unweighted UniFrac; full data set, PERMANOVA pseudo-F = 1.95, BH *P* = 0.008; MSM data set, PERMANOVA pseudo-F-statistic = 2.076, BH *P* = 0.003), and coinfected independent individuals had higher alpha diversity than dependent individuals (Faith’s PD; full data set, K-W H = 7.79, BH *P* = 0.0053; MSM data set, K-W H = 8.523, BH *P* = 0.0035).

**FIG 1:**
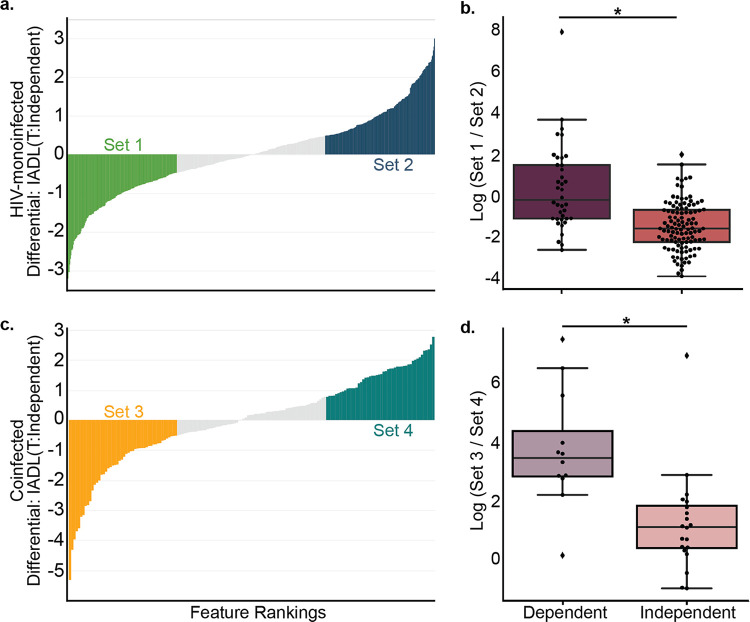
Feature rankings and differentials derived from Songbird (a and c) and boxplots of the log ratios of the taxa sets (b and d). (a and b) HIV-monoinfected (HIV+/HCV−) MSM subgroup. (c and d) Coinfected (HIV+/HCV+) MSM subgroup. *, In panel b, * = *t* test *P* = 3.35e−10, *t* = −6.74. In panel d, * = *t* test *P* = 0.00062, *t* = −3.82.

Conserved community-wide microbiome differences according to IADL status within both the HIV-monoinfected and coinfected (HIV+/HCV+) full cohorts and MSM subgroups prompted us to identify taxa enriched in dependent individuals. We computed the log ratio of the highest 30% (“Set 1” in Table S1) and lowest 30% (“Set 2” in Table S1) of the ranked suboperational taxonomic units [sOTUs] associated with IADL status in the HIV-monoinfected MSM subgroup using Songbird (12), visualized with Qurro (13) (see “Methods” below). We performed the same analysis using the coinfected MSM subgroup and computed the log ratio of the highest 30% (“Set 3” in Table S1) and lowest 30% (“Set 4” in Table S1) of ranked sOTUs associated with IADL status in the coinfected MSM subgroup. Both cases revealed significant differences in log ratios of features distinguishing independent from dependent individuals (HIV-monoinfected individuals, Fig. 1a and b, *t* test *P* = 3.35e−10, *t* = −6.74; coinfected individuals, Fig. 1c and d, *t* test *P* = 0.00062, *t* = −3.82). The HIV-monoinfected and coinfected feature sets had shared sOTUs: set 1 and set 3 had 8 sOTUs in common, and set 2 and set 4 had 17 sOTUs in common. The same feature sets were also used successfully to distinguish IADL-dependent from -independent individuals in the full HIV-monoinfected and coinfected groups (HIV-monoinfected group, *t* test *P* = 8.0e−06, *t* = −4.85; coinfected group, *t* test *P* = 0.0032, *t* = −3.25). Although identification of particular microbes associated with these disease states is limited, of interest is *Bacteroides* which has been previously associated with mild cognitive impairment and lower global cognitive function (14), present in 8.1% (15/186 total features) in set 1 but only 3.2% (6/186) in set 2 and 12.5% (6/48) in set 3 but 0% (0/48) in set 4, suggesting that *Bacteroides* is enriched in IADL-dependent people in both the HIV-monoinfected and coinfected infection groups. The proportion of *Bacteroides* sOTUs in the unselected feature space was 2.4% (6/251) and 3.0% (2/66) for HIV-monoinfected and coinfected MSM subgroups, respectively. Since IADL may be plausibly linked to sexual practice, and the ratio of *Bacteroides* to *Prevotella* has been reported to be associated with sexual practice, changes in *Bacteroides* should be interpreted with caution. Notably, because *Bacteroides* can be decreased in the human gut by increasing carbohydrate intake and/or reducing animal protein (15), dietary changes may prevent or treat microbiome-based cognitive decline in these populations.

10.1128/mSystems.00528-20.1 TABLE S1 Sets of unique taxa identified from Songbird and used in log ratio calculations. Download Table S1, CSV file, 0.1 MB. Copyright © 2020 Taylor et al. 2020 Taylor et al. This content is distributed under the terms of the Creative Commons Attribution 4.0 International license.

10.1128/mSystems.00528-20.2 TABLE S2 Metabolomics beta diversity (Bray-Curtis) PERMANOVA results. Download Table S2, CSV file, 0.01 MB. Copyright © 2020 Taylor et al. 2020 Taylor et al. This content is distributed under the terms of the Creative Commons Attribution 4.0 International license.

### Methods.

**(i) Participant recruitment, sample processing, and sample selection.** This was a cross-sectional prospective observational cohort study of persons with or without HIV infection recruited from community sources, who agreed to undergo comprehensive neuromedical and neurobehavioral evaluations for NIH-funded studies at the HIV Neurobehavioral Research Program (HNRP) (https://hnrp.hivresearch.ucsd.edu/) including the HIV Neurobehavioral Research Center (HNRC) study (study details, HNRP [16]) at the University of California San Diego (UCSD). Those who also agreed to submit stool samples for microbiome studies were included in the current analyses. A subset of participants also had positive serology for hepatitis C virus. The UCSD’s Human Research Protections Program (irb.ucsd.edu) approved all study procedures, and all participants provided written informed consent (UCSD institutional review board [IRB] protocol number 172092).

Exclusions were diagnoses of active substance use disorders and presence of an active, major psychiatric condition with current psychotic features or neurological conditions such as schizophrenia or epilepsy. If multiple stool samples were collected from participants, only the first time point was analyzed by 16S rRNA sequencing. A single time point per subject was additionally analyzed by high-performance liquid chromatography coupled to tandem mass spectrometry (HPLC-MS/MS). HIV and HCV infection was confirmed by a point-of-care vertical flow test (MedMira, Halifax, Nova Scotia). Participants were designated (i) “HIV monoinfected” if they tested positive for HIV but not HCV, (ii) “coinfected” if they tested positive for both HIV and HCV, or (iii) “uninfected” if they tested positive for neither HIV or HCV.

**(ii) Psychiatric assessment.** Psychiatric and substance use diagnoses were obtained using the Composite International Diagnostic Interview (CIDI) (www.hcp.med.harvard.edu/wmhcidi/), a fully structured, computer-based interview, to determine DSM-IV diagnoses for current and lifetime mood and substance use disorders. DSM-IV diagnosis of lifetime and current major depressive disorder was evaluated using CIDI. Current self-reported depressed mood was assessed using the Beck Depression Inventory-II. The BDI-II consists of 21 items that assess the severity of depression symptoms over the 2 weeks prior to assessment. The BDI-II total score ranges from 0 to 63 with higher scores denoting more severe depression symptoms. For analyses, we used the published cutoff of at least mild severity to define current self-reported depression. Parameters of lifetime substance use were estimated using a semistructured timeline follow-back interview (TLFBI) as previously described (17). The TLFBI uses a calendar method to evaluate daily patterns and frequency of substance use over a specified time period. It has high retest reliability, convergent and discriminant validity with other measures, agreement with collateral informants’ reports of patients’ substance use, and agreement with results from patients’ urine assays.

**(iii) Assessments of measured activities of daily living.** Dependence in instrumental activities of daily living (IADLs) was assessed with a modified version of the Lawton and Brody Scale that asks participants to rate their current and best lifetime levels of independence for 13 major IADLs such as shopping, financial management, transportation, and medication management. An employment questionnaire asked about the following: job loss; decreases in work productivity, accuracy, and quality; increased effort required to do one’s usual job; and increased fatigue with the usual workload.

**(iv) Neuromedical and laboratory assessment.** All participants underwent a comprehensive neuromedical assessment, including a medical history that collected antiretroviral therapy (ART) and other medications, data to determine Centers for Disease Control and Prevention (CDC) staging, and specimen collection (blood, stool). Routine clinical chemistry panels, complete blood counts, rapid plasma reagin, and CD4^+^ T cells (flow cytometry) were performed at a Clinical Laboratory Improvement Amendments (CLIA)-certified medical center laboratory. HIV RNA was measured in plasma using reverse transcriptase PCR (Amplicor, Roche Diagnostics, Indianapolis, IN) with a lower limit of quantitation of 40 copies/ml.

**(v) 16S rRNA gene sequencing.** DNA extraction and 16S rRNA amplicon sequencing were done using Earth Microbiome Project (EMP) standard protocols (http://www.earthmicrobiome.org/protocols-and-standards/16s). DNA was extracted with the Qiagen MagAttract PowerSoil DNA kit as previously described (18). Amplicon PCR was performed on the V4 region of the 16S rRNA gene using the primer pair 515f to 806r with Golay error-correcting barcodes on the reverse primer. Amplicons were barcoded and pooled in equal concentrations for sequencing. The amplicon pool was purified with the MO BIO UltraClean PCR cleanup kit and sequenced on the Illumina MiSeq sequencing platform. Sequence data were demultiplexed and minimally quality filtered using the Qiita defaults.

**(vi) 16S marker gene data analysis.** QIIME 2 v2020.2 (19) was used to rarefy to 2,500 sequences/sample and to generate pairwise unweighted UniFrac distances. Between-group differences based on these distances were tested using PERMANOVA and permuted *t* tests in QIIME 2. Alpha diversity (Faith’s PD) was compared with a Kruskal-Wallis test.

Songbird v1.0.1 (12) in QIIME 2 version 2020.2 was used to identify feature ranks (parameters: –p-epochs 10000 –batch-size 5 –learning-rate 1e-4 –min-sample-count 1000 –min-feature-count 0 –num-random-test-examples 10% of samples), and Qurro v0.4.0 (13) was used to compute log ratios of these ranked features. Evaluation of the Songbird models against a baseline model obtained a pseudo-Q2 value of >0, suggesting that the models were not overfit, except for the coinfected full group model which had a pseudo-Q2 of −0.048 and suggests possible overfitting related to the subtlety of the differences between IADL states in coinfected individuals when not accounting for sexual preferences. *t* tests were calculated to assess the significance (alpha = 0.05) of the log ratios.

**(vii) LC-MS/MS data acquisition.** Data acquisition protocols follow the Center for Microbiome Seed Grant Metabolomics standards, as performed in the study of Taylor et al. (9). Human fecal samples were transferred to clean 2-ml sample tubes (Qiagen catalog no. 990381), and the weights were recorded. The samples were then extracted in a 1:1 solution of methanol to water spiked with an internal standard of 1 μM sulfamethazine, using a 1:10 sample weight (milligram) to solvent volume (microliter) ratio. Using a Tissuelyser II (Qiagen), the samples were homogenized for 5 min at 25 Hz. This was followed by a 15-min centrifugation at 14,000 rpm. From the supernatant, 400 μl was transferred to a prelabeled 96-well DeepWell plate, and the plates were concentrated using a CentriVap benchtop vacuum concentrator (Labconco) for approximately 4 h. The dry plates were placed into the −80°C freezer until time for analysis.

The plates were resuspended in 150 μl of a 1:1 solution of methanol to water with a 1 μM sulfadimethoxine internal standard solution. For metabolomics analysis, an ultrahigh performance liquid chromatography system (Thermo Dionex Ultimate 3000 UHPLC) coupled to an ultrahigh resolution quadrapole time of flight (qToF) mass spectrometer (Bruker Daltonics MaXis HD). For chromatographic separation, a Phenomenex Kinetex column (C_18_; 1.7 μm, 2.1 mm by 50 mm) was used. The mobile phase consisted of solvent A (100% LC-MS grade water with 0.1% formic acid) and solvent B (100% acetonitrile with 0.1% formic acid). Each sample was injected at a volume of 5 μl into a flow rate of 0.5 ml for the entire analysis. The 12-min chromatographic gradient began at 5% solvent B for the first minute, an increase to 100% solvent B from minute 1 to minute 11, a hold at 100% solvent B until minute 11.5, and back down to 5% solvent B reached at minute 11.5. All data were collected using electrospray ionization in positive mode. Positive mode was selected in order to allow for spectral matches to be found using the Global Natural Products Social Molecular Networking (GNPS) spectral libraries, a majority of which were collected in positive ionization mode. Data-dependent acquisition was set to a scan range of 100 to 2,000 *m/z*.

**(viii) LC-MS/MS data analysis.** The raw data in Bruker (.d) format were lock mass corrected using hexakis ((1H, 1H, 2H-difluoroethoxy)phosphazene (Synquest Laboratories, Alachua, FL) and were exported as .mzXML files using the Bruker Data Analysis software. Both the raw .d and the .mzXML files were uploaded to the UC San Diego mass spectrometry data repository MassIVE (https://massive.ucsd.edu/ProteoSAFe/static/massive.jsp). Feature detection was completed using MZmine version 2.37 software (20). The resulting feature tables were exported as both a quantification file (.csv) and a spectral information file (.mgf) for analysis using the Global Natural Products Social Molecular Networking platform (21).

The quantification table and spectral information were analyzed using the GNPS feature-based molecular networking workflow. Parameters can be viewed via the job results page (https://gnps.ucsd.edu/ProteoSAFe/status.jsp?task=350392e8e24c41f2b84fde04f9183fc4). Also available at this job result page is access to the molecular networks and library annotations for the entire data set. This data set consisted of 1,911 unique MS/MS spectra, of which 313 had a spectral match to the GNPS reference libraries (https://gnps.ucsd.edu/ProteoSAFe/libraries.jsp), including matches to drug metabolites, bile acids, food-related compounds, and dipeptide molecules. For the statistical analyses, the MZmine-produced feature abundance table containing peak areas was input into the web-based MetaboAnalyst software. The data were normalized following the metabolomics data analysis protocols outlined in the previous metabolomics project (22), a normalization by quantile normalization and an auto scale. The normalized data were used to calculate a squareform matrix based on the Bray-Curtis distance metric which was input into a .qza format for use in QIIME 2. All PERMANOVAs were run using the QIIME 2 beta group significance command (19). Individual feature level comparisons were completed using a Dunn’s test.

**(ix) Data availability.** The data generated in this study are available publicly in Qiita under the study identifier (ID) 11135 (https://qiita.ucsd.edu/study/description/11135), and sequence data associated with this study have been deposited at EBI/ENA under accession number ERP122366. The GNPS feature-based molecular networking job is available at https://gnps.ucsd.edu/ProteoSAFe/status.jsp?task=350392e8e24c41f2b84fde04f9183fc4. The raw experimental data are available at MassIVE (https://massive.ucsd.edu/), data set MSV000083664.
